# Phantom Arm Pain and Tinnitus in a Patient with ST-Segment Elevation Myocardial Infarction: A Case Report

**DOI:** 10.5811/cpcem.47095

**Published:** 2025-12-15

**Authors:** John Porter, Chad P. Liedl, Kevin P. Steever, Hunter D. Lumby, Chase M. Hanson, Hannah Rae R. Vaden, Aaron B. Klassen

**Affiliations:** * Mayo Clinic, Cardiac Catheterization Laboratory, Rochester, Minnesota; † Mayo Clinic, Ambulance Service, Rochester, Minnesota; ‡ Mayo Clinic, Division of Audiology, Rochester, Minnesota; § Mayo Clinic, Department of Emergency Medicine, Rochester, Minnesota

**Keywords:** arm pain, case report, phantom limb pain, STEMI, tinnitus

## Abstract

**Introduction:**

We present the case of a patient with the unusual occurrence of phantom arm pain and an acute exacerbation of chronic tinnitus during an ST-segment elevation myocardial infarction (STEMI).

**Case Report:**

A 56-year-old man was having several classic symptoms associated with acute coronary syndrome, along with perceived pain in an arm lost years earlier in a traumatic accident and a sudden worsening of his chronic tinnitus. Emergency medical services responded and diagnosed a STEMI on scene. A 100% occlusion of his right coronary artery was rapidly identified in the hospital and treated with the deployment of two drug-eluting stents. After the procedure his symptoms resolved. He was discharged without incident two days later.

**Conclusion:**

Whereas arm pain is a well-documented presenting symptom of acute coronary syndrome, phantom limb pain and exacerbation of tinnitus have been rarely reported in the literature.

## INTRODUCTION

The diagnosis of acute coronary syndrome is not always straightforward. Although chest pain is the hallmark symptom, it can be accompanied by a spectrum of atypical symptoms. These can include nausea, vomiting, dyspnea, radiation of pain to the arm or jaw, diaphoresis, fatigue, lightheadedness, back pain, headache, otalgia, sore throat, hand pain, and anxiety, among others.^1^–^4^ Up to 44% of patients with non-ST-segment elevation myocardial infarction (STEMI) and 27% of patients with STEMI do not have chest pain.^5^ Our case describes a patient with STEMI who presented with phantom limb pain and exacerbation of chronic tinnitus.

## CASE REPORT

A 56-year-old man with a history of acute coronary syndrome, obesity, and obstructive sleep apnea presented with worsening left arm pain. The patient had sustained a left upper extremity amputation up to his shoulder from a motor vehicle accident years prior. He also was a tobacco user and experienced chronic tinnitus and neuropathy in his lower extremities. Approximately 10 years earlier the patient underwent a cardiac stress test after having an episode of chest pain that he described as like “being punched in the chest.” This led to a hospital admission and a stress test at an outside facility, although he was unsure of the details of the stress test, and no records could be located. No interventions were performed or medications prescribed.

He had been experiencing chronic and unchanging paresthesias in his phantom limb since the amputation. On the day of presentation, a sudden onset of chest pain and pressure developed in the center of his chest, which he described as sharp and rated 7 (on a scale of 0–10). The pain radiated to his right arm and left shoulder. There was also substantial worsening of the chronic paresthesia in his left upper phantom limb, which he described as “someone trying to pull my arm off.” The chest pain decreased to 4 (on a scale of 0–10) after lying down, but he then became lightheaded, dyspneic, diaphoretic, pale, clammy, and reported “feeling weird.” In addition, he noted an “extreme” increase in his baseline tinnitus. He then had a syncopal episode witnessed by his significant other who contacted emergency medical services (EMS).

**Image 1:**
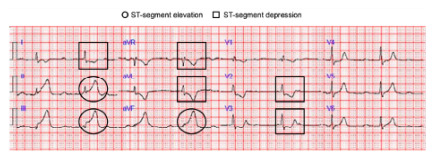
The ECG indicates ischemia as a secondary consequence of the patient’s myocardial infarction. The circles highlight ST segment elevation in the inferior leads, while the squares mark ST segment depression in the lateral and anterior leads.

Electrocardiography obtained by EMS showed a STEMI (Image 1), and the cardiac catheterization lab was alerted from the field. Initial vital signs obtained by EMS revealed the following: blood pressure, 129/73 millimeters of mercury; heart rate, 110 beats per minute; respiratory rate, 32 breaths per minute; and oxygen saturation 94% on room air. Electrocardiography performed on arrival to the emergency department showed a worsening elevation in the inferior leads and atrial fibrillation. His troponin T value measured by Elecsys Troponin T Gen 5 STAT (Roche Diagnostics Corporation, Indianapolis, IN) was 608 nanograms/Liter (ng/L) (reference range: ≤ 15 ng/L).

**Image 2:**
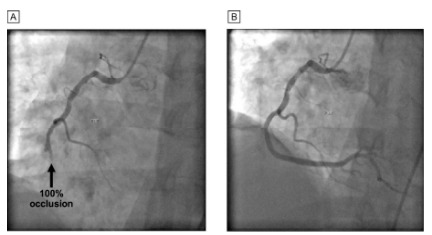
Angiography performed in the cardiac catheterization lab: A, right coronary artery before intervention, and B, right coronary artery after intervention.

He was found to have 100% occlusion of his distal right coronary artery, which was treated with two drug-eluting stents (Image 2). After percutaneous coronary intervention, his symptoms resolved, including improvement in his phantom limb paresthesia to baseline. During his hospital stay he was newly diagnosed with diabetes and hyperlipidemia and was discharged two days after admission. On follow-up, the patient continues to do well after significant lifestyle modifications and management of his chronic diseases.

## DISCUSSION

This case is unique in that it illustrates yet another atypical symptom that can be present in a patient experiencing acute coronary syndrome. A lack of chest pain does not rule out a STEMI. Literature suggests that 20–30% of patients experiencing an episode of acute coronary syndrome have no chest pain at all.^6^, ^7^ Pain in the left arm is a known symptom of an MI.^8^ Stimuli arising in the myocardium during an acute coronary syndrome episode activate the same area in the brain that is activated by signals from somatic structures such as the arm. Arm pain is thought to arise from a convergence of inputs from cardiac and somatic inputs in the trigeminal nucleus located in the brainstem.^9^ Misinterpretation of these two inputs by the brain leads to the referred pain.^8^

*CPC-EM Capsule*

What do we already know about this clinical entity?

*The underlying pathophysiology and constellation of presenting symptoms in ST-segment elevation myocardial infarction (STEMI) are well defined.*

What makes this presentation of disease reportable?

*We discuss a rare presenting symptom of arm pain in a phantom limb, along with a worsening of chronic tinnitus, in a patient with STEMI.*

What is the major learning point?

*Patients experiencing an episode of acute coronary syndrome may not present with symptoms more commonly associated with STEMI.*

How might this improve emergency medicine practice?

*This case highlights uncommon presenting symptoms in a patient with STEMI.*

Left-arm phantom pain, however, a symptom not previously seen by any member of the patient’s care team, is an uncommon symptom of an MI. The literature describing such a phenomenon is limited. Martin et al^10^ described a 49-year-old man with exertional chest pain that radiated into a phantom limb; the pain resolved after the patient had coronary artery bypass grafting surgery. Cohen and Jones^11^ reported two instances of patients with chest and phantom left-arm pain accompanied by evidence of damage to the myocardium.

Tinnitus is an awareness of sound with no external auditory stimuli and is usually a symptom of hearing loss or a change in hearing.^12^ A weak association between tinnitus and heart disease has been reported.^13^ Tinnitus has been correlated with a higher incidence of cardiac events but not of death due to cardiovascular disease.^14^ Arterial stiffness, which is a known predictor of cardiovascular events and mortality,^15^ is significantly associated with both the development and severity of tinnitus.^16^ Stress also is known to be related to the intensity of tinnitus symptoms. Neuroplasticity as an adaptation to stress is a key component in the progression of tinnitus,^17^ and stressful events can lead to a worsening of tinnitus.^18^, ^19^ We postulate that the increased stress, both conscious and subconscious, caused by the STEMI led to the worsening of our patient’s tinnitus from the chronic baseline level.

## CONCLUSION

This case highlights the unusual symptoms that can manifest during a STEMI and provides a telling anecdotal report of the impact that rapid treatment, along with lifestyle changes, can have on a patient’s quality of life.
